# Structure and function of a short LOV protein from the marine phototrophic bacterium *Dinoroseobacter shibae*

**DOI:** 10.1186/s12866-015-0365-0

**Published:** 2015-02-14

**Authors:** Stephan Endres, Joachim Granzin, Franco Circolone, Andreas Stadler, Ulrich Krauss, Thomas Drepper, Vera Svensson, Esther Knieps-Grünhagen, Astrid Wirtz, Anneliese Cousin, Petra Tielen, Dieter Willbold, Karl-Erich Jaeger, Renu Batra-Safferling

**Affiliations:** Institute of Molecular Enzyme Technology, Heinrich-Heine-Universität Düsseldorf, Forschungszentrum Jülich, D-52425 Jülich, Germany; Institute of Complex Systems, ICS-6: Structural Biochemistry, Forschungszentrum Jülich, D-52425 Jülich, Germany; Juelich Centre for Neutron Science JCNS (JCNS-1) & Institute for Complex Systems (ICS-1), Forschungszentrum Jülich, D-52425 Jülich, Germany; Institute for Microbiology, Technische Universität Braunschweig, D-38106 Braunschweig, Germany; Institute of Physical Biology, Heinrich-Heine-Universität Düsseldorf, D-40225 Düsseldorf, Germany; Institute of Bio- and Geosciences, IBG-1: Biotechnology, Forschungszentrum Jülich, D-52425 Jülich, Germany

**Keywords:** Aerobic anoxygenic photosynthesis, Blue-light photoreceptor, Crystal structure, Dimerization, *Dinoroseobacter shibae*, LOV domain, PAS domain, Photocycle

## Abstract

**Background:**

Light, oxygen, voltage (LOV) domains are widely distributed in plants, algae, fungi, bacteria, and represent the photo-responsive domains of various blue-light photoreceptor proteins. Their photocycle involves the blue-light triggered adduct formation between the C(4a) atom of a non-covalently bound flavin chromophore and the sulfur atom of a conserved cysteine in the LOV sensor domain. LOV proteins show considerable variation in the structure of N- and C-terminal elements which flank the LOV core domain, as well as in the lifetime of the adduct state.

**Results:**

Here, we report the photochemical, structural and functional characterization of DsLOV, a LOV protein from the photoheterotrophic marine α-proteobacterium *Dinoroseobacter shibae* which exhibits an average adduct state lifetime of 9.6 s at 20°C, and thus represents the fastest reverting bacterial LOV protein reported so far. Mutational analysis in *D. shibae* revealed a unique role of DsLOV in controlling the induction of photopigment synthesis in the absence of blue-light. The dark state crystal structure of DsLOV determined at 1.5 Å resolution reveals a conserved core domain with an extended N-terminal cap. The dimer interface in the crystal structure forms a unique network of hydrogen bonds involving residues of the N-terminus and the β-scaffold of the core domain. The structure of photoexcited DsLOV suggests increased flexibility in the N-cap region and a significant shift in the Cα backbone of β strands in the N- and C-terminal ends of the LOV core domain.

**Conclusions:**

The results presented here cover the characterization of the unusual short LOV protein DsLOV from *Dinoroseobacter shibae* including its regulatory function, extremely fast dark recovery and an N-terminus mediated dimer interface. Due to its unique photophysical, structural and regulatory properties, DsLOV might thus serve as an alternative model system for studying light perception by LOV proteins and physiological responses in bacteria.

**Electronic supplementary material:**

The online version of this article (doi:10.1186/s12866-015-0365-0) contains supplementary material, which is available to authorized users.

## Background

Signal perception in LOV domains is achieved *via* a blue-light (λ = 440–482 nm) absorbing flavin mononucleotide (FMN) or, in some cases, *via* a flavin adenine dinucleotide (FAD) chromophore, that in the dark is non-covalently bound within the LOV domain [1,2]. Blue-light absorption triggers the formation of a transient adduct between the FMN-C4a atom and the sulfur atom of a strictly conserved cysteine residue in the LOV domain active site [1]. Once illumination has ceased, this covalent bond is broken thus concluding the photocycle. While the initial photochemical reaction and the adduct formation occurs on a timescale of microseconds [3], adduct decay can take seconds to hours depending on the LOV protein [3-7] (Additional file 1: Table S1). The longest living intermediate, the FMN-cysteinyl-thiol adduct, has been proven to represent the structural signaling state of LOV photoreceptors [8,9]. The majority of LOV photoreceptors are multi-domain sensory systems, where light-signal perception by the LOV domain influences the “activity” of fused effector domains such as kinases, anti-sigma factors, helix-turn-helix DNA binding domains, phosphodiesterases and cyclases [10].

Based on this modularity, LOV photoreceptors in turn control or influence a multitude of cellular light responses in plants, algae, fungi and bacteria [11]. In plants, the biological function of LOV photoreceptors is well-established, including plant phototropism, chloroplast relocation, light-induced leaf movement and regulation of stomatal opening [12,13]. In contrast, the understanding of regulatory functions of their counterparts in bacteria is still limited, some examples were described recently (comprehensively reviewed in [14]). The best characterized bacterial LOV photoreceptor is YtvA from the soil bacterium *Bacillus subtilis* [15], which is part of the stressosome, involved in the σ^B^-mediated general stress response of this organism [9,16-18]. Recently, additional regulatory functions of bacterial LOV proteins were described such as control of cell-adhesion in *Caulobacter cresentus* [19], virulence in *Brucella abortus* [20], motility of *Pseudomonas syringae* [19,21,22] and in the counteraction of plant immune response during citrus canker in *Xanthomonas citri* [23].

Remarkably, in addition to the multi-domain LOV photoreceptors, a large subset of LOV photoreceptors exist in bacteria and fungi that do not possess fused effector domains and are thus called “short” LOV proteins. Functionally and structurally characterized examples include the fungal photoreceptor VVD [7], the LOV proteins PpSB1-LOV and PpSB2-LOV of *Pseudomonas putida* KT2440 [4,5,24] and the recently characterized RsLOV protein of *Rhodobacter sphaeroides* 2.4.1 [25,26]. Physiological studies on *Neurospora crassa* VVD and recent data for RsLOV indicate that despite the lack of a fused effector domain, short LOV proteins can act as genuine blue-light receptors, probably transducing the signal *via* interactions with a partner protein.

All short LOV proteins structurally characterized so far possess N- and/or C-terminal helical extensions (N-terminal N-cap or A´α-helix, and C-terminal Jα-helix) outside the conserved LOV core domain suggested to be involved in signaling [7,24,26]. In multi-domain LOV photoreceptors, these extensions often link the sensor LOV and associated effector domains and are suggested to act as signal-transducing linker elements or spacers providing flexibility [13,27].

Another feature common to most LOV domains is their dimerization propensity. In particular, a light-dependent alteration of the monomer-dimer equilibrium represents a feasible mode of signal-relay for short LOV proteins, where one of the two quaternary structural states selectively mediates the interaction with downstream signaling partners [25,28,29]. For short LOV proteins crystallized as full-length constructs, dimerization is mostly mediated by either the N-terminal A´α-helix (N-cap) and/or, if present, the respective C-terminal Jα-helices [24,25,28]. The presence and structure of N- and C-terminal extensions largely determine the mode of dimerization and hence the corresponding light-dependent conformational change. Thus, the study of short LOV proteins with unusual LOV-core extensions might provide further insights into the diversity of LOV-protein dimerization and signaling.

Here, we report on the identification of an unusual short LOV protein DsLOV from the marine phototrophic α-proteobacterium *Dinoroseobacter shibae* DFL12^T^. Combining crystallography and biochemical methods, we investigate the molecular mechanism of photoexcitation in DsLOV, which displays a recovery on the timescale of seconds. Furthermore, we demonstrate that the fast recovering DsLOV is a key regulator for light mediated triggering of photopigments bacteriochlorophyll *a* and spheroidenone formation in *D. shibae*.

## Results and discussion

### *Dinoroseobacter shibae* harbors three putative LOV photoreceptor genes

**Figure 1:**
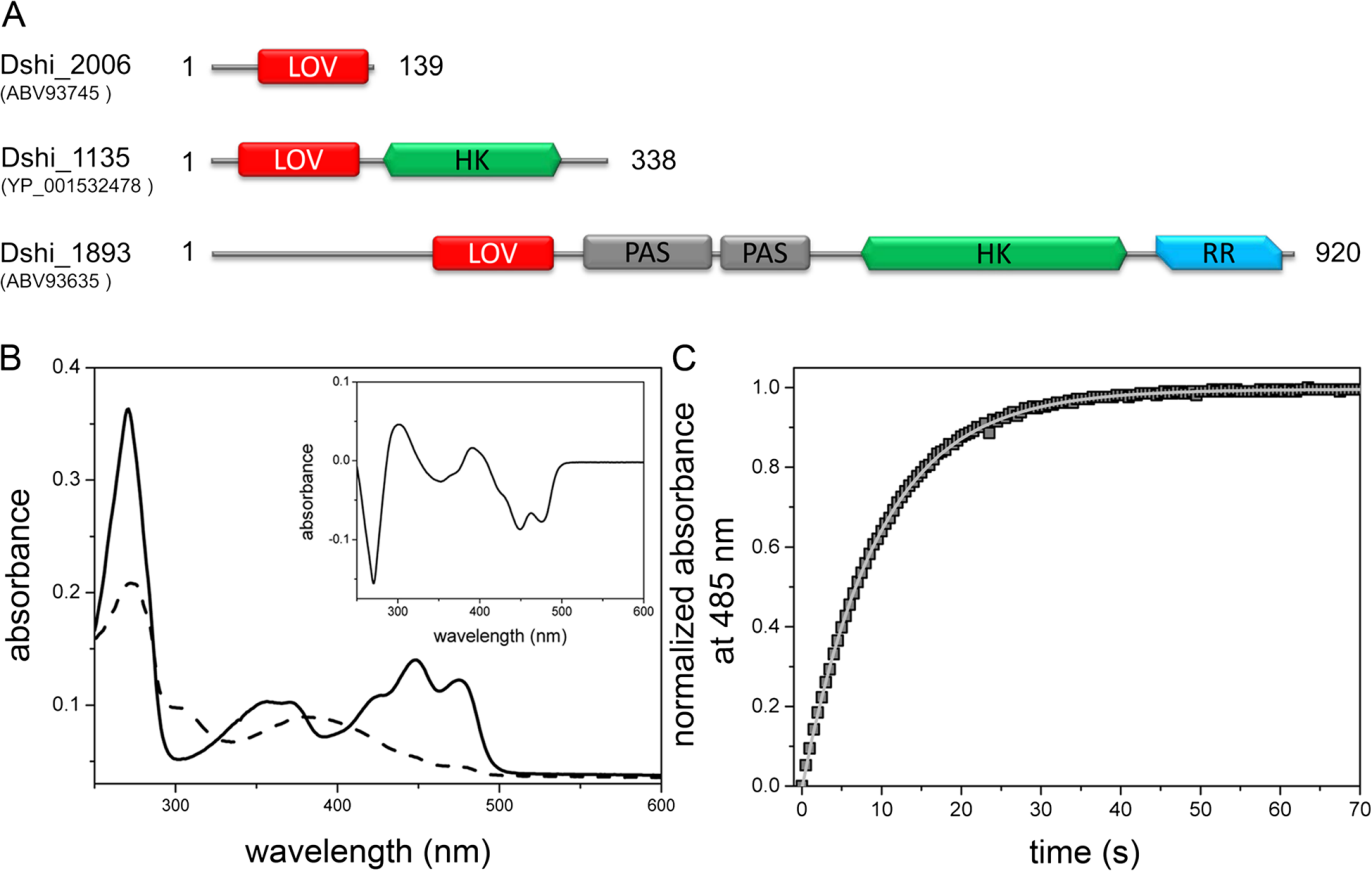
**DsLOV domain architecture and photochemistry. (A)** Domain architecture of the three *D. shibae* LOV proteins. Locus tags and accession numbers (in brackets) are given in front of each protein. The length of each protein and the type and relative location of all predicted Pfam domains are specified. LOV: light, oxygen voltage; HK: histidine kinase; PAS: PER-ARNT-SIM; RR: response regulator. **(B)** Absorbance spectra of DsLOV in the dark and light state. In the dark, DsLOV displays the characteristic UV/vis absorbance bands with a λ_max_ of about 449 nm (solid line). To sufficiently populate the light state, the DsLOV protein was exposed to blue-light for 30 seconds at 4°C (dashed lines), resulting in a decreased absorbance at 447 nm and the concomitant formation of a new absorbance maximum at 390 nm. The inset shows the light–dark difference spectrum of DsLOV. **(C)** Dark recovery of DsLOV measured at 20°C. LOV protein dark recovery was monitored by recording time traces for the absorbance recovery at 485 nm after blue-light illumination. The data (squares) was fitted using a mono-exponential decay function.

The Gram-negative α-proteobacterium *Dinoroseobacter shibae* is a member of the *Roseobacter* clade, a bacterial lineage that is globally abundant in different marine habitats. Light is an important environmental factor for *D. shibae* because it can produce ATP *via* aerobic anoxygenic photosynthesis [30,31]. Sequence similarity analysis of the published *D. shibae* DFL12^T^ genome revealed the presence of three genes encoding putative blue-light photoreceptors of the LOV family (Additional file 1: Figure S1), possessing all the sequence features necessary for FMN binding and photocycling [1]. The predicted LOV proteins encoded by the genes Dshi_1135 and Dshi_1893 show either one single histidine kinase domain or four domains with homologies to PAS, histidine kinase and response regulator protein domains, which are fused to the respective C-termini of the predicted LOV domains (Figure 1A). In contrast, the gene Dshi_2006 codes for a short LOV protein that lacks fused effector domains. Among bacterial short LOV proteins, DsLOV appears exceptional as it harbors an N-terminal cap domain but lacks a C-terminal extension and is thus architecturally very similar to fungal VVD [7].

### DsLOV exhibits fast recovery kinetics

The soluble photoreceptor protein DsLOV was expressed in *E. coli* BL21 (DE3) as C-terminal His_6_-tagged protein and was purified *via* immobilized-metal affinity chromatography (IMAC). In the dark, DsLOV exhibits the characteristic absorption bands of the LOV dark state with λ_max_ = 449 nm (Figure 1B, solid lines). Remarkably, formation of the DsLOV photoadduct (λ_max_ = 390 nm) after blue-light illumination, reflected by the loss of its absorption within the blue-region of the spectrum could only be observed when the sample temperature was reduced to 4°C (Figure 1B, dashed lines) suggesting a rapid dark recovery which was determined as τ_REC_ = 9.6 ± 0.1 s at 20°C (Figure 1C).

### Crystal structure of DsLOV reveals a unique N-cap region

#### Overall structure

**Figure 2:**
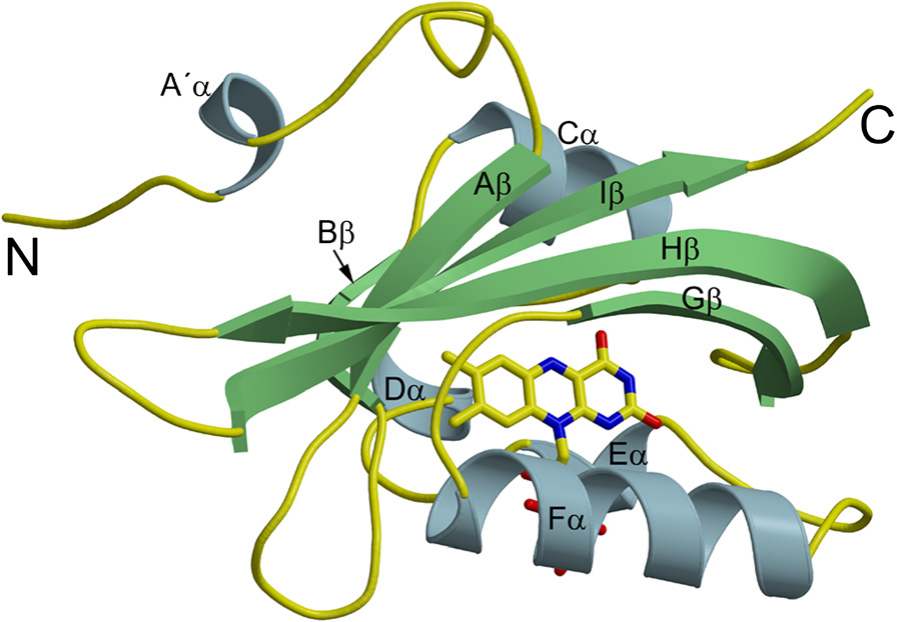
**Crystal structure of DsLOV.** Ribbon representation of the dark state of DsLOV (residues 20–139) colored according to secondary structure elements with helices in light blue; β-strands in light green, and loops in yellow). The chromophore riboflavin (RBF) is shown as stick model and is colored by element: carbon, yellow; nitrogen, blue; oxygen, red.

Monoclinic crystals of the DsLOV protein were obtained in the dark, which diffracted to a resolution of 1.5 Å. The statistics of X-ray data collection and refinement are documented in Table 1. The final monomer model (one molecule per asymmetric unit) comprises residues 20–139 of the protein and the chromophore riboflavin (RBF) (Figure 2; the secondary structure elements are colored with helices in light blue; β-strands in light green, and loops in yellow; the chromophore is shown as stick model and is colored by element: carbon in yellow, nitrogen in blue and oxygen in red). Residues 1–19 could not be traced in the electron density map and are likely to be disordered. Also, no electron density was observed for the C-terminal residues 139–146 that comprise the His_6_-tag. According to Ramachandran plot generated with Molprobity [32], the model exhibited good geometry with none of the residues in disallowed regions.

**Table 1:** **Data collection and refinement statistics**

	**DsLOV “dark”**	**DsLOV “photoexcited”**
Wavelength (Å)	0.93340 (Beamline: ESRF, ID14-1)	0.95372 (Beamline: ESRF, ID23-1)
Detector type	ADSC Quantum Q210	ADSC Quantum Q315r
Resolution range (Å)	45.4 - 1.5 (1.53 - 1.5)^a^	26.1 - 2.0 (2.07 - 2.0)^a^
Space group	C 1 2 1	C 1 2 1
Unit cell	a = 89.98 (Å)	a = 90.78 (Å)
	b = 30.60 (Å)	b = 30.94 (Å)
	c = 49.33 (Å)	c = 49.54(Å)
	β = 113.03°	β = 113.09°
Total reflections	65088	30165
Unique reflections	19912	8206
Multiplicity	3.3 (2.5)	3.7 (4.0)
Completeness (%)	98.98 (91.59)	93.48 (98.15)
Mean I/σ (I)	19.7 (4.3)	16.3 (10.4)
Wilson B-factor (Å^2^)	14.08	10.62
R-sym	0.037 (0.203)	0.061 (0.085)
R-work	0.1298 (0.1115)	0.1633 (0.1595)
R-free	0.1789 (0.2031)	0.1824 (0.1586)
Coordinate error (maximum-likelihood based) (Å)	0.13	0.16
Number of atoms	1125	1015
Macromolecules	981	904
Ligands	32	27
Water molecules	112	84
Protein residues	120	115
RMS (bonds) (Å)	0.014	0.004
RMS (angles) (°)	1.56	0.77
Ramachandran favored (%)	99	99
Ramachandran outliers (%)	0	0
Clash score	2.99	4.92
Average B-factor (Å^2^)	19.9	14.7
PDB entry	4KUK	4KUO

^a^Statistics for the highest-resolution shell are shown in parentheses.

The residues 36–137 comprise the conserved LOV core domain with a typical α/β-fold PAS topology. The core domain is made up of a five-stranded antiparallel β-sheet comprising Aβ (residue 36–40), Bβ (residue 49–52), Gβ (residue 98–105), Hβ (residue 111–122) and Iβ (residue 128–137), and four α-helices Cα (residue 54–60), Dα (residue 64–67), Eα (residue 72–75) and Fα (residue 82–94). In addition, a noncanonical structural element designated as the N-cap is present that includes a N-terminal turn (residues 20–23), helix (A´α, residues 24–27) and a connecting loop (28–35) (Figure 2).

### Dimer interface

**Figure 3:**
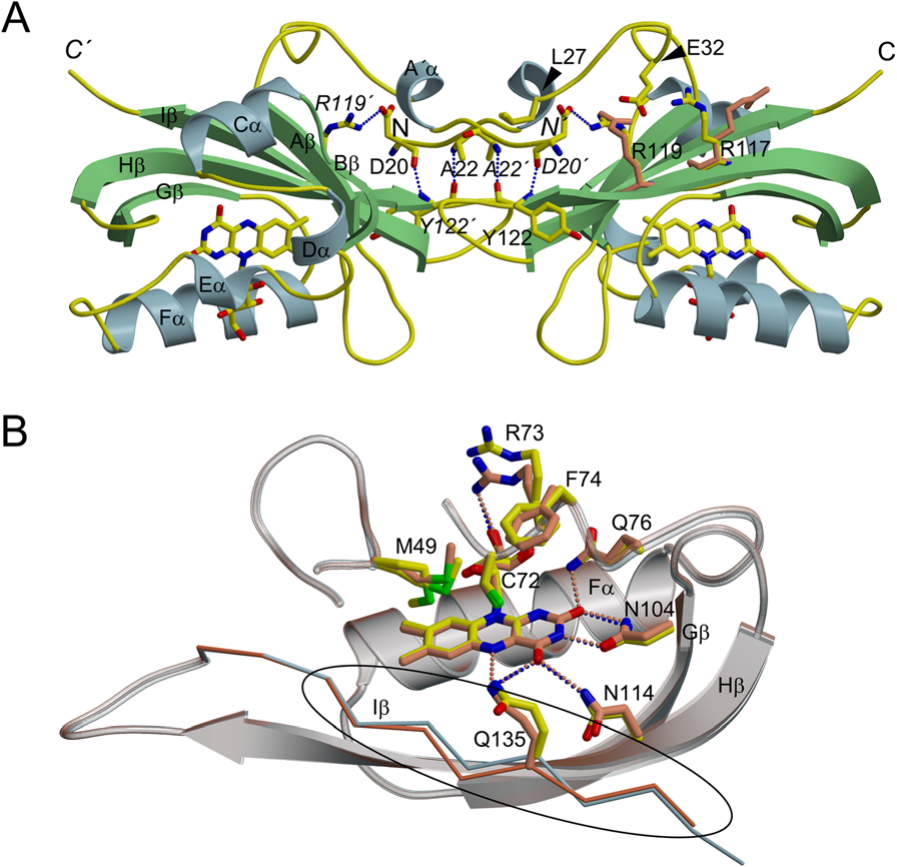
**Dimer interface and photoexcited state of DsLOV. (A)** The dimer of DsLOV (dark state) showing the N-terminus (A´α) mediated dimer interface, based on PISA analysis [33]. The secondary structure elements are colored with helices in light blue; β strands in light green, and loops in yellow. The chromophore riboflavin (RBF) is shown as stick model and is colored by element: carbon, yellow; nitrogen, blue; oxygen, red. The intersubunit hydrogen bonds are shown as broken lines between the residues shown as stick models and colored by element. Specifications on the hydrogen bond distance and the atoms involved are provided in the text. Side chain conformations of R117 and R119 in photoexcited state are shown superimposed in coral. Position of L27 and E32 are indicated by arrowheads. **(B)** Superposition of dark and photoexcited crystal structures. Side chains of residues shown as stick models are colored by element for dark state as in A, and in coral for photoexcited state. A shift seen in the respective Iβ backbone (dark state- light blue and photoexcited- coral) is highlighted in the oval area. For clarity, rest of the region is shown as ribbon representation in transparent grey. Note the additional H-bond between Q135-NE2 and N5 of RBF molecule in the photoexcited state. The view in this figure is of the monomer (right) of the dimer shown in panel A, which is rotated ~ 180° around the horizontal axis.

The DsLOV crystal structure has one molecule in the asymmetric unit even though the protein exists as a dimer in solution as revealed by SEC and small angle X-ray scattering experiments described below. The most probable higher-order assembly proposed by PISA analysis [33] is a dimer, where the N-terminal A´α helix is part of the dimer interface that is unique amongst LOV protein structures (Figure 3A). The buried surface area of ~950 Å^2^ involves a set of interchain interactions between charged residues (D20-OD2…*NH1*-*R119*´, 3.4 Å) (residues in italics with prime refer to those from the symmetry equivalent molecule), hydrogen bonds (D20-O…*N*-*Y122*´, 2.7 Å, A22-N…*O*-*Y122*´, 2.8 Å) and several hydrophobic interactions between residues in the N-terminal cap (including helix A´α) and the β-scaffold of the opposite subunit (Hβ and Iβ) (Figure 3A). Residues constituting the dimer interface are I21, L24, I50, L68, G69, I121, D123, P124, E125, M129 and F130 from the two-fold related monomers.

### Structural comparison with other LOV domains

Although the LOV core domain is structurally well conserved, diversity is seen for the noncanonical N- and C-terminal elements [7,24,26,34,35]. These regions adopt the helical secondary structure in the previously published LOV protein structures (Additional file 1: Figure S2). An N-terminal turn-helix-turn motif has been previously reported [34] in the crystal structure of AsLOV2 (PDB ID 2V0U, yellow in Additional file 1: Figure S2). In DsLOV, the N-terminal extension shows similar secondary structure elements with a turn, helix and a long connecting loop (Figure 2). However, superposition of the core domains (Additional file 1: Figure S2) shows different positioning of the corresponding N-terminal elements. In contrast to monomeric AsLOV2, the noncanonical N-terminal element of DsLOV is involved in dimer formation. We next compared DsLOV to fungal VVD [28] as both proteins consist of the conserved core domain with an N-terminal extension. Surprisingly, despite the differences in N-cap length (DsLOV: 35 and VVD: 71 aa long) and secondary structure, superposition of the VVD dark state (PDB ID 2PD7, orange in Additional file 1: Figure S2, panel B) shows the highest similarity, where residues 43–50 and 67–72 follow the trace corresponding to the N-cap residues of DsLOV. Moreover, N-cap mediated dimerization is globally similar in both structures. Functional implications of this similarity are discussed below in the section ‘light-induced conformational changes’.

### Flavin binding pocket

As seen in previously reported dark state LOV protein structures, a flavin chromophore molecule is noncovalently bound within a tight pocket formed by the β-scaffold and the surrounding Eα- and Fα-helices (Figure 2). Surprisingly, the chromophore bound in the DsLOV structure is RBF and not FMN; where both are identical except for the terminal phosphate group that is absent in RBF. Prior to crystallization setups, purified DsLOV protein was denatured and the released chromophores were analyzed by HPLC demonstrating that it predominantly binds FMN (74.3 ± 0.2%) and FAD (25.7 ± 0.2%) whereas no riboflavin could be detected (data not shown). We suspect that the FMN bound to the DsLOV protein in solution got hydrolyzed to RBF during the crystallization time period of several weeks in acidic conditions [36].

The mode of protein-chromophore interaction is conserved amongst the LOV protein family, where rigid coordination of the chromophore molecule in the binding pocket is mediated by a network of hydrogen bonds, van der Waals and electrostatic interactions. Mainly, residues in the 3_10_ Eα-helix (N71, R73, Q76), Hβ-strand (N104, N114), and Iβ strand (Q135) constitute most of the hydrogen bonding interactions with the RBF molecule. Residues forming hydrophobic contacts with the isoalloxazine ring are V38, C72, I88, R89, L92, L116, I118, F131, A132 and G133.

In all LOV proteins, two conserved arginine residues make contact with the phosphate group of the chromophore. In DsLOV, the corresponding arginines are R73 and R89, where only R73 forms a hydrogen bond (R73-NH2…O5´-RBF, 3.0 Å) with the ribityl chain. Please note that the interaction between the terminal end of chromophore ribityl chain and the protein is most likely disturbed due to presence of RBF (and not FMN) in the DsLOV crystal structure, hence preventing any conclusions with regard to the FMN-phosphate coordination in DsLOV in solution, where FMN is the predominant chromophore (see above results of HPLC analysis).

### Light-induced conformational changes

The photochemistry of LOV domains is reversible in solution as well as in crystals [7,34,35,37]. We thus solved the structure of DsLOV in the photoexcited state after illuminating crystals grown in the dark (Table 1). The difference electron density map (F_dark_-F_light_) does not show the presence of a covalent bond between residue C72 and flavin C4a atom (Additional file 1: Figure S3, panels A-B). The interatomic distance between the C72-Sγ and RBF-C4a is 3.44 Å, marginally smaller than 3.54 Å observed in the DsLOV dark state structure. The presence or absence of a covalent adduct in LOV protein crystals has been used in previous studies to define either the ‘light’ or ‘dark’ state, respectively. The absence of a covalent adduct in the photoexcited crystal structure of DsLOV can be due to two reasons: (i) fast dark recovery of the protein (τ_REC_ = 9.6 s) prevents adduct stabilization, and/or (ii) radiation damage with radiolysis of the Cys72-RBF thioether bond caused by the high-energy X-ray beam. The latter has been previously observed for other LOV proteins [7,34,37]. Additionally, comparison of the spectra obtained from single crystal microspectrometry confirmed typical dark state (λ_max_ = 447 nm) and light state (λ_max_ ≈ 390 nm) absorbance spectra for the DsLOV crystals grown in dark, and after blue-light illumination, respectively (Additional file 1: Figure S4, panel A). In order to verify that spectral changes observed in the crystal are not the result of irreversible photobleaching, dark grown DsLOV crystals were transferred to the microspectrometer under constant blue-light illumination in the cryo stream (100 K) and an absorbance spectrum was recorded. Subsequently, the cryo stream was blocked and the light was switched off to allow dark recovery of the protein at room temperature. The corresponding spectra shows full recovery of the flavin specific absorption band in the blue-region of the spectrum within 5 minutes (Additional file 1: Figure S4, panel B). In contrast, no dark recovery occured within 1 hour at cryogenic temperatures (Additional file 1: Figure S4, panel C). Taken together, the data demonstrates that the protein in dark grown DsLOV crystals is capable of reversible photocycling, suggesting that the structural changes observed in photoexcited crystals are most likely the result of illumination and are not related to irreversible photobleaching.

Several distinct conformational differences between dark and photoexcited DsLOV structures were observed, even though direct evidence supporting an adduct formation in the electron density map is absent in the latter (Figure 3B): (i) several residues primarily localized at the boundary of the chromophore binding pocket show different side chain conformations in the photoexcited state; these include V37, V38, M49, E65, V67, R70, R73, F74, R97, R117, R119, I121 and E125; (ii) due to absence of electron density, residues 29–33 located in the A´α-Aβ loop could not be traced in the photoexcited state; (iii) a significant shift is observed in the Cα backbone of the Iβ strand (~0.7 Å Cα - Cα distance) (Figure 3B), the Aβ strand (~0.6 Å), and the connecting loop (A´α-Aβ loop in the N-terminus) (Additional file 1: Figure S3, panels C-D); (iv) the hydrophobic pocket around the dimethyl-benzene ring of the chromophore is also rearranged; for example, A132 and G133 no longer make hydrophobic contacts as in the dark state. An interesting observation is that the side chain conformation of F74 which is aligned parallel to the flavin ring system in the dark rotates towards the flavin ring in the photoexcited state (Figure 3B). Notably, we observed similar side chain orientations for this structurally conserved phenylalanine residue in previously published crystal structures of *C. reinhardtii* Phot LOV1 (PDB ID 1N9L, 1N9N, 1N9O), *B. subtilis* YtvA LOV (PDB ID 2PR5, 2PR6), *R. sphaeroides* RsLOV (PDB ID HJ6, 4HNB) and *P. putida* PpSB1-LOV (PDB ID 3SW1). Mutation of the corresponding phenylalanine residue in YtvA-LOV to histidine accelerated the dark recovery of the protein 25-fold [38]. This hints a possible involvement of F74 in photoactivation, signaling and adduct state stability in LOV proteins and further suggests that the structural differences observed between dark and photoexcited structures of DsLOV are a consequence of photoactivation.

Despite the increased flexibility in the N-terminus (absence of electron density for residues 29–33 located in the A´α-Aβ loop) in the photoexcited state, residues 20–28 were still traceable (Additional file 1: Figure S3, panel C). The position of the N-cap, in particular, is stabilized by hydrogen bonding interactions between dimer-interface residues D20-*Y122*´, and A22-*Y122*´, that exist in both the dark- and photoexcited-states. It should be noted that the photoexcited state structure was obtained upon illumination of dark-grown crystals, which is not sufficient to cause large conformational changes of functional relevance that would require breaking of the crystal lattice constraints. Interestingly, the side chain conformation of R119, involved in the dimer interface differs in both the states, facing towards and away from the dimer interface in the dark and photoexcited state, respectively (Figure 3A). As a result, interactions between R119 and *D20*´, R119 and L27, and R119 and E32 are absent. Energetically, this might increase the flexibility of the N-terminus as seen in the photoexcited state (disordering of the A´α-Aβ loop). Another major structural change is observed for the side chain conformation of R117 in the photoexcited state. Here, R117 forms a salt bridge with N136 on Iβ (R117-NH1…OD1-N136, 2.49 Å and R117-NH2…OD1-N136, 2.71 Å). In the corresponding dark state structure, the side chain of R117 is flipped towards the N-terminal cap (Figure 3A) breaking the interaction with N136. Residue Q135, that forms a single hydrogen bond with the RBF isoalloxazine ring – O4…NE2-Q135, 3.07 Å, shows two possible hydrogen bonds O4…NE2-Q135, 2.93 Å, and N5…NE2-Q135, 3.02 Å in the photoexcited state (Figure 3B) (Additional file 1: Figure S3, panel B). Hydrogen bonding between the conserved glutamine and the newly protonated N5 atom of the flavin ring has been observed in other light state crystal structures [24,35,37,39]. This light-induced structural change of Q135 is one of the initial steps in LOV photoactivation that probably contributes to the observed displacement of the Iβ strand in the photoexcited state of DsLOV.

In VVD, structures of dark-grown crystals are monomeric [7]. Both, solution studies and light state crystal structure (obtained from crystals grown under light conditions) show homodimer formation [28]. Structural rearrangements in the latter include release of the N-terminal ‘latch’, which then binds to the symmetry equivalent molecule allowing dimer formation. In contrast, the N-terminus is packed against the β-sheet of the LOV core domain in structures obtained from dark-grown crystals. An important role in the process is played by the hinge region that connects the N-terminus and the core domain, and shows structural changes as a result of FMN-cysteinyl-thiol adduct formation. Interestingly, the corresponding region in DsLOV (residues 28–35 in A´α-Aβ loop) shows no electron density upon photoexcitation. Considering the analogy between DsLOV and VVD that show N-cap mediated dimer interface, it is possible that the mode of interaction as well as signal transfer are globally similar in both proteins where an illumination-triggered relay of structural changes occurs from the chromophore binding pocket towards the N- and C-terminus, gated *via* residues on the central β-scaffold (Iβ and Hβ). These might involve change in conformation of the A´α-Aβ loop in the N-cap region, which could consequently enable signal-transduction to an as yet unknown downstream partner protein of DsLOV within the cellular context.

### Structural basis of the fast dark recovery of DsLOV

Most positions in the vicinity of the flavin chromophore known to influence the adduct decay such as V38 (V416 on Aβ in AsLOV2 [40,41]), N104 and N114 (N94 and N104; YtvA numbering, [42]) which form hydrogen-bonds to the isoalloxazine C(4) = O and C(2) = O atoms, as well as N47 on the Aβ-Bβ loop (N37, YtvA numbering, [43]) are conserved between DsLOV and other LOV proteins, and are thus less likely to be involved in accelerating the dark recovery of DsLOV compared to other LOV proteins. Additionally, it has been previously suggested that adduct decay can be influenced by surface exposed histidine residues by base-catalysis *via* a proposed hydrogen-bonding network of the base, the chromophore and intraprotein water molecules [3,44]. While DsLOV possesses one surface exposed histidine (H83) about 18 Å away from the RBF-C4a atom (H83-NE2…C4a-RBF, 18.5 Å), no direct or indirect proton relay network can be identified in the structure, ruling out a significant impact of the respective residue on the fast recovery of DsLOV.

One major structural difference in the vicinity of the flavin chromophore between DsLOV and other plant and bacterial LOV domains is the presence of a methionine residue (M49) on Bβ at the dimethylbenzene side of the flavin isoalloxazine ring. In other LOV domains, the corresponding position is either occupied by an isoleucine (YtvA, AsLOV2, VVD) [40] or leucine (PpSB1-LOV) [24]. For AsLOV2, YtvA and VVD, mutation of the respective isoleucine residue strongly influences the adduct-state lifetime. Here, the mutations I427V (AsLOV2), I39V (YtvA) and I85V (VVD) accelerated the recovery of the respective proteins 8-, 5- and 23-fold [40,45]. Several structural interpretations have been proposed for the observed mutation-induced kinetic effects. For AsLOV2, I427V was proposed to be in van der Waals contact with the sulfhydryl group of the photoactive cysteine (C450) [45]. The authors suggested that structurally, I427 provides steric support to the active site region near the photoactive cysteine and that removal of this support by introducing a valine at the respective position destabilizes the adduct state and hence results in a faster dark recovery. Moreover, different imidazole-base dependent adduct-decay rate accelerating effects were observed for VVD and the corresponding I85V mutant. In particular, the adduct-decay rate acceleration was more pronounced for VVD-I85V than for the wild-type protein. This suggests better access of imidazole to the active site of VVD-I85V where it acts as a base to abstract the proton from N5 proton in the light state, hence accelerating the photocycle dark recovery [40]. Taken together, residue-alterations at the respective position on Bβ seem to influence the dark recovery of LOV proteins by both steric effects acting on the photoactive cysteine and by controlling solvent/base access to the active site, which drives adduct scission by N5 deprotonation.

In the DsLOV dark-state crystal structure, M49 possesses two conformations (Additional file 1: Figure S3, panel A), one where the δ-sulfur atom of the methionine side-chain is facing towards C72-Sγ (C72-SG…SD-M49, 3.43 Å) and a second mirrored conformation where the δ-sulfur is facing away from the photoactive cysteine (C72-SG…SD-M49, 3.89 Å). The double occupancy of M49 is indicative of structural flexibility of the respective residue in the dark. Even though we do not directly observe C72-Sγ - FMN-C4a bond formation in photoexcited crystals of DsLOV, adduct formation would result in a movement of C72-Sγ towards the RBF-C4a atom and hence away from M49. For reversal of the reaction, the C72-Sγ – RBF-C4a bond has to be broken and C72 has to move back towards M49. Consequently, this back movement might be eased by the flexibility of M49 in the dark state of DsLOV, whereas it would be more restricted in the presence of a branched amino acid such as isoleucine in AsLOV2, VVD and YtvA, thus resulting in a slower dark recovery of the latter proteins compared to DsLOV. Likewise, M49 might act as a “gate-keeper” restricting/enabling the solvent/base access to FMN-N5 hence influencing adduct decay by N5 deprotonation as suggested for VVD [40].

### Small angle X-ray scattering reveals dimeric DsLOV in solution

**Figure 4:**
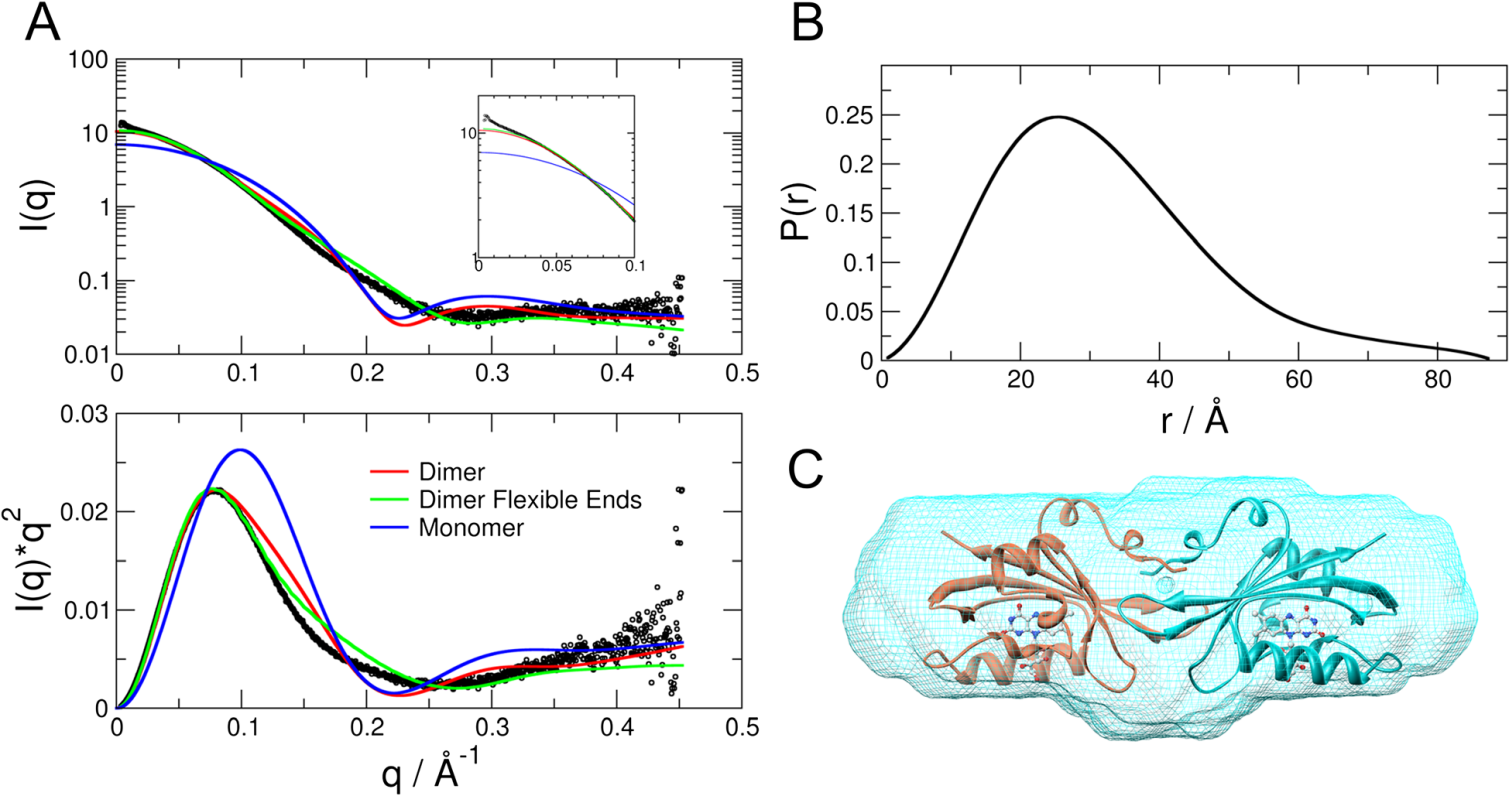
**Solution behavior of DsLOV measured by small angle X-ray scattering. (A)** Experimental data (open circles), calculated curves of the crystal structure of the monomer (blue line), the crystal structure of the dimer (red line), and the crystal structure of the dimer including flexible ends (green line). The inset shows the zoom in on the low q-range, where a small fraction of aggregated particles cause the slight uptick at q < 0.024 Å^−1^. The lower panel shows the same experimental data and the fits in a Kratky-plot. **(B)** The distance distribution function P(r) of DsLOV as calculated for the range of q > 0.024 Å^−1^, where effects of protein aggregation are negligible. **(C)** *Ab initio* model of DsLOV (cyan, shown in mesh) determined by SAXS and aligned crystal structure of the dimer (dimer orientation as in Figure 3A, two protein molecules are shown in coral and cyan with bound RBF molecules as stick model).

We performed SAXS measurements to determine the oligomeric state and envelope of the molecule in solution. The molecular mass determined from the Porod volume and *ab initio* modelling was 33.4 kDa and 33.7 kDa, respectively. The calculated molecular mass from the amino acid sequence of monomeric DsLOV is 16.85 kDa. Analytical size-exclusion chromatography (SEC) revealed an apparent molecular mass of 35.6 ± 0.5 kDa for DsLOV, corresponding to the calculated mass of the dimer (33.7 kDa) (data not shown). Therefore, both SEC and SAXS results demonstrate that the protein forms a dimer in solution. Next, theoretical scattering curves were calculated for the monomer and the dimer of the crystal structure, and fitted against the experimental SAXS data. As shown in Figure 4A, the theoretical curve of the monomer is a poor fit that yields a large *χ*^2^-value of 47. A better fit with dimer results in a reduction of *χ*^2^-value to 11.9, however, this is still significantly large. Notably, the dimer fits well in the small scattering vector range up to 0.1 Å^−1^ whereas deviations become visible at larger *q*-values. Scattering in the small angle region is sensitive to the molecular mass and to the global shape, whereas the large *q*-region is more sensitive to the tertiary structure of the protein. The positions of the first 19 N-terminal residues and the C-terminal His_6_-tag are not resolved in the crystal structure. Disorder and flexibility of the missing regions was thus accounted for by modelling these regions in the crystal structure of DsLOV dimer using the EOM program [46]. The resulting theoretical curve produces the best fit with a reduced *χ*^2^-value of 5.8 (Figure 4A). The discrepancy of the theoretical curve calculated from the dimer structure is thus related to the missing terminal regions.

The envelope of DsLOV was determined by *ab initio* modelling. The distance distribution function P(r) is shown in Figure 4B. The *ab initio* model obtained using program GASBOR combined with the aligned crystal structure of the dimer is shown in Figure 4C. The general shape of the envelope confirms the dimeric state of the protein and overlaps the dimer structure. In fact, the non-filled parts of the envelope correspond to the locations of the crystallographically non-resolved residues in the N- and C-terminus.

### DsLOV is involved in the upregulation of photopigment synthesis in the absence of blue-light

The facultative phototrophic bacterium *D. shibae* can grow either heterotrophically in the dark or photoheterotrophically in the light by using a photosystem that contains the photopigments bacteriochlorophyll (BChl) *a* and the carotenoid spheroidenone [47]. In contrast to anoxygenic photosynthetic α-proteobacteria, *D. shibae* synthesizes its photopigments in the presence of oxygen [30,48]. However, since photoexcited BChl *a* efficiently transfers energy to molecular oxygen which in turn leads to the formation of toxic reactive oxygen species (ROS), *D. shibae* down regulates the synthesis of photopigments upon illumination [30,31,49].

**Figure 5:**
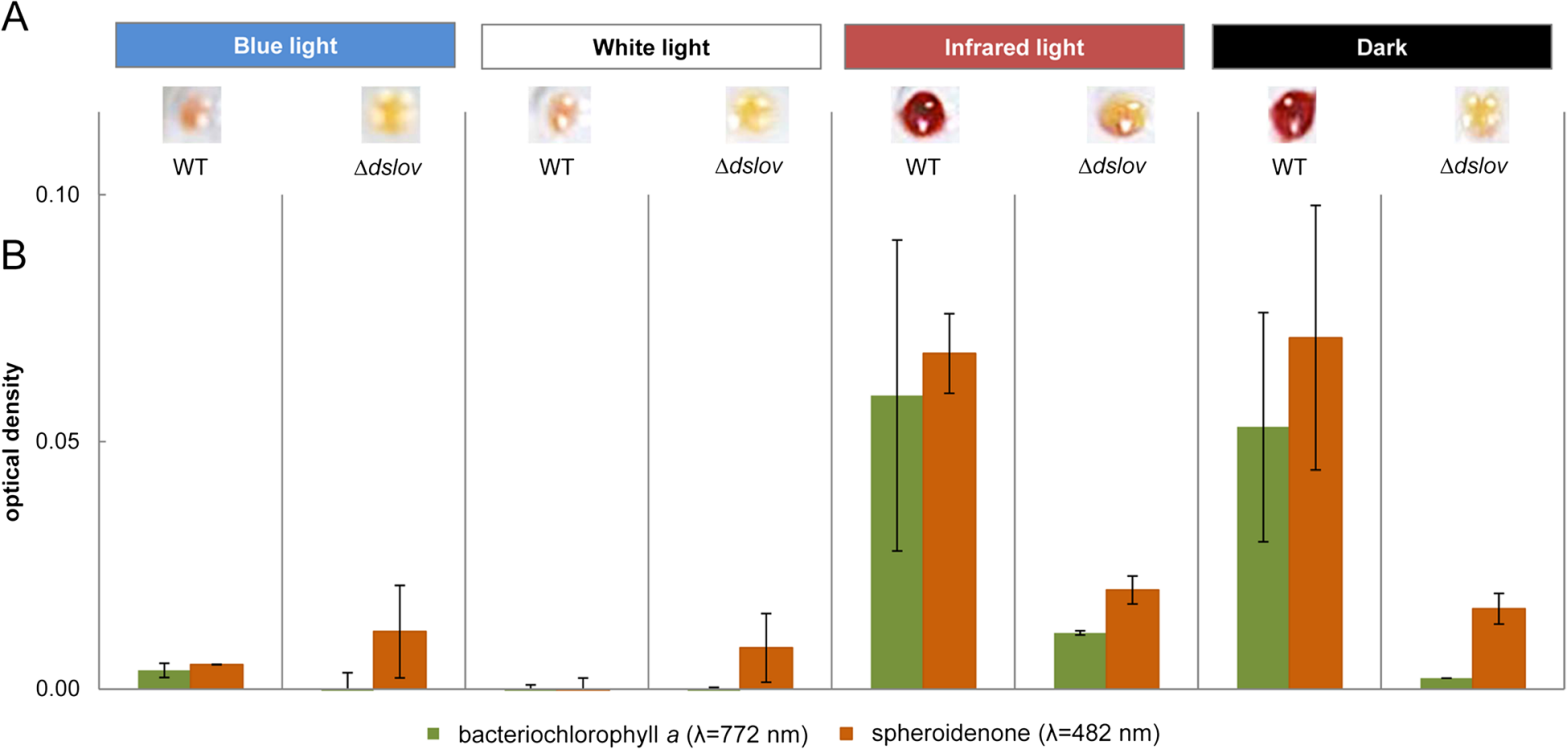
**Characterization of a DsLOV-deficient** ***D*** **.** ***shibae*** **mutant strain. (A)** Influence of different light regimes on pigment composition of *D. shibae* wild-type (WT) and the DsLOV-deficient mutant strain (Δ*dslov*). Strains were grown in the dark, under white-, infrared-, and blue-light conditions until the cells reached the late logarithmic growth phase. After harvesting 1 ml of cell culture by centrifugation, cell pellets were photographed to document the individual pigmentation. **(B)** Spectroscopic determination of light-dependent photopigment composition in *D. shibae* wild-type and mutant Δ*dslov*.

We first analyzed the effect of blue-light on the accumulation of BChl *a* and spheroidene in *D. shibae* to determine if the photoreceptor DsLOV is involved in light-dependent control of photopigment formation. For this, we comparatively cultivated *D. shibae* cells heterotrophically in the dark as well as photoheterotrophically using three different light sources for illumination, namely (i) daylight emulating neon tubes emitting broad spectrum light of wavelengths from 400 nm to 720 nm; (ii) infrared-light (IR) LEDs exhibiting a maximal emission at 855 nm (half-maximal width 837–864 nm) and (iii) blue-light LEDs maximally emitting at 462 nm (half-maximal width 455–477 nm). These specific emission wavelengths were chosen because they are suitable for the excitation of BChl *a* (*in vivo* absorption in the blue and infrared range of the light spectrum) and spheroidenone (absorption of blue-light). After cultivation, differences in photopigment accumulation were analyzed (Figure 5A).

As expected, significant amounts of photopigments accumulated in *D. shibae* wild-type strain in the dark indicated by a deep red coloration of the corresponding cell pellet. In contrast, illumination with broad-spectrum light led to an almost complete loss of coloration (Figure 5A). Interestingly, blue-light illumination of *D. shibae* wild-type cells resulted in a similar down regulation of pigment synthesis, whereas no inhibitory effect was observed upon infrared-light irradiation. In this case, cells showed a red coloration that had so far only been observed for dark grown cells.

To quantitatively analyze the pigment composition of dark-grown and illuminated cells, photopigments were extracted and the absorption of spheroidenone and BChl a (λ_max_ of isolated photopigments: 482 nm for spheroidenone and 772 nm for BChl a) were determined as described in the methods section (Figure 5B). Dark grown and infrared illuminated cells showed comparable absorption values for both photopigments. In contrast, the absorption values of photopigments extracted from white or blue-light irradiated *D. shibae* cells were negligible indicating that neither BChl *a* nor spheroidenone accumulated under these conditions. These observations indicate that photopigment synthesis in *D. shibae* is predominantly controlled by a blue-light signal provided here by the blue-light LEDs or by the predominant blue part of the white-light spectrum.

We next analyzed if the blue-light photoreceptor DsLOV is involved in this regulatory process. We constructed a *D. shibae* DsLOV deletion strain (*D. shibae* Δ*dslov*) and subsequently analyzed pigment accumulation under the illumination conditions described above. The results clearly demonstrate that the Δ*dslov* strain did not show any coloration, regardless of the applied illumination conditions (Figure 5A, B) demonstrating that induction of pigment formation in the DsLOV-deficient *D. shibae* strain was almost completely abolished under all tested light regimes.

Our data suggest that photopigment synthesis in *D. shibae* is primarily controlled by blue-light with the short LOV photoreceptor DsLOV acting as a key regulator. Based on the presented observations, we propose a unique regulatory function where the dark state of DsLOV seems to be the regulatory relevant state which is directly or indirectly involved in the activation of photopigment synthesis.

As shown for other aerobic anoxygenic phototrophic bacteria (AAPB), *D. shibae* performs anoxygenic photosynthesis in the presence of light and oxygen to gain additional ATP. However, since light-excitation of BChl *a* in the presence of molecular oxygen can result in the formation of cytotoxic reactive oxygen species (ROS) including singlet oxygen (^1^O_2_) [50], it might be of vital importance for *D. shibae* to control photopigment synthesis in response to changing light regimes. Accordingly, Tomasch and coworkers could demonstrate by transcriptome analysis that the transition from dark to light conditions resulted in an induction of ROS-mediated stress response accompanied by a down-regulation of photosynthesis genes [30]. Remarkably, based on our finding, the photoreceptor DsLOV might act as a ROS-independent dark-sensor which is essential for induction of pigment formation under non-hazardous illumination conditions. In accordance with this assumption, light-mediated inhibition of photopigment synthesis was only provoked by blue light whereas infrared light that is particularly absorbed by Bchl *a* is irrelevant for the observed regulatory process. In this context, it is worth mentioning that infrared light is rapidly attenuated in water and thus does not penetrate a water column further than ca. 5 m, whereas blue light can reach a water depth of more than 30 m. Because *D. shibae* is a marine bacterium which lives in symbiosis with *dinoflagellates* in coast-near aquatic habitats [47], blue light might be the most sensitive environmental factor for the proposed adaptation process.

In contrast to the light regulated photopigment synthesis in *D. shibae,* expression of photosynthesis genes in the facultative photosynthetic bacterium *R. sphaeroides* is predominantly regulated by oxygen tension. High oxygen concentration leads to activation of the repressor molecule PpsR which strictly prevents the expression of photosynthesis genes [51,52]. However, a second blue-light triggered regulatory mechanism comes into play under micro-aerobic conditions where the blue-light photoreceptors AppA and CryB bind to the repressor PpsR in the dark to inactivate its suppressing function [53-56]. Also, the short LOV blue-light receptor RsLOV is involved in the light-mediated control of photosynthesis related genes [25,26,56]. An RsLOV mutant strain exhibited a stronger pigmentation and a higher BChl *a* content during the late exponential phase. Furthermore, RsLOV effects the gene expression of photooxidative stress response genes as well as genes for chemotaxis and carbohydrate metabolism [26]. Very recently, it was published that the regulator AerR acts as an antirepressor of the PpsR homolog CrtJ in the closely related phototrophic bacterium *R. capsulatus*, which inhibits the CrtJ-mediated repression of photosynthesis genes in a vitamin B12-dependent and light-responsive manner [57,58]. Despite missing information about the detailed mode of intracellular signal transduction, our functional data suggests that photosynthesis pigment formation is apparently controlled very differently in *D. shibae* than in other closely related photosynthetic microbes, even though similar blue-light responsive photoreceptors are employed.

## Conclusions

The photochemical and structural characterization of the ‘short’ DsLOV protein highlights some unique properties including a very fast recovery, a flexible N-terminal cap region as well as a dimeric structure in solution stabilized mainly *via* an unusual set of interactions at the molecule interface. Based on the differences between the crystal structures of the dark state and the photoexcited state, we identified several key residues in the chromophore binding pocket which play an important role in signaling and are located in the hinge region connecting the N-cap to the LOV core domain. These results lead us to postulate that in DsLOV subtle light-induced structural changes in the chromophore binding pocket are subsequently propagated, *via* several residues located in the Iβ and Hβ strands of the central β-scaffold, towards the peripheral regions and manifested as partially disordered loop in the N-cap region. However, transfer of the light signal in DsLOV might require conformational changes at a larger scale than observed here as permitted by the crystal lattice. Additionally, we have demonstrated that DsLOV is essential for the induction of photopigment formation in the dark whereas blue-light specifically leads to almost complete downregulation of Bchl *a* and spheroidenone accumulation.

The results of our functional studies show that photopigment synthesis in *D. shibae* takes place only in the dark when DsLOV is likely to be in a dimeric state. Compared to the crystal structure of the dark state dimer, the DsLOV photoexcited state shows several structural rearrangements including an increase in flexibility in the N-cap region involved in dimer formation. It is thus tempting to speculate that these blue-light mediated structural changes (that might eventually be more pronounced in solution) could lead to a quaternary structural change of the protein. This in turn could result in altered protein-protein interactions inside the cell, which would enable the reported physiological response.

Its unique photophysical properties and regulatory functions, as well as the distinct and quantifiable phenotype of a mutant, make DsLOV a new and promising model system to study the intramolecular and regulatory processes of LOV photoreceptors.

## Methods

### Bacterial strains and growth conditions

Bacterial strains were cultivated as batch cultures in sterilized Erlenmeyer flasks with a culture to vessel volume ratio of at least 1:5. *E. coli* strains were grown in LB-medium (Carl Roth, Karlsruhe, Germany) at 37°C under continuous shaking at 130 rpm. *D. shibae* strains were cultivated under equal shaking conditions in marine bouillon medium (Carl Roth, Karlsruhe, Germany) at 30°C. Depending on the plasmid mediated resistance, culture media were supplemented with kanamycin (50 μg/ml), ampicillin (100 μg/ml) or gentamycin (100 μg/ml). Aminolevulinic acid (ALA, 50 μg/ml) was added to *E. coli* ST18 cultures. Light-conditioned experiments were performed in darkness, infrared, blue and white-light. For the blue and infrared illumination, a set of previously described light emitting diodes (LED)-arrays were used [59]. Each of the two LED panels contain 120 blue LEDs (NSSB100BT, λ_max_ = 462 nm, Nichia, Japan) and 120 high power infrared LEDs (SFH 4257, λ_max_ = 855 nm, Osram, Munich, Germany). The average photon flux at a distance of approximately 12 cm from the LED panel was as follows: blue-light (462 nm): 55 μmol m^−2^ s^−1^, Infra-red light (855 nm): 43 μmol m^−2^ s^−1^. White-light illumination was carried out with daylight emulating neon tubes ‘865 Lumilux Cool Daylight’ (Osram, Munich, Germany). Cell cultures were placed between the light sources at a constant distance of 12.5 cm on each side.

### Cloning of DsLOV

First, a 423-bp DNA fragment encompassing the DsLOV gene without (i) the first ATG codon at the 5′-end encoding one of two annotated N-terminal methionine residues and (ii) its stop codon, was PCR-amplified by using the primers DsLOV + *NdeI*-up (5′-GAGTCGCATATGCGCAGACATTATCGCGACCTGAT-3′) and DsLOV + *XhoI*-Stop-dn (5′-AATAATCTCGAGGACCGGCTTCTGGGCGCCTGCGAAGAA-3′) as well as the genomic DNA of *Dinoroseobacter shibae* DFL12^T^ as template DNA. The PCR fragment was ligated into the *SmaI*-site of pBluescript KS (+). Next, the recombinant plasmid pBluescript-DsLOV was hydrolyzed with *Nde*I and *Xho*I to isolate the 419-bp DsLOV DNA fragment which was subsequently cloned into the respective sites of the T7 expression vector pRhotHi-2 [60]. The final DsLOV expression vector was designated as pRhotHi-DsLOV. The correct sequences for all vectors described in the manuscript were verified by DNA sequencing.

### Construction of a *D. shibae* ∆*dslov* strain

The *D. shibae dslov* (dshi_2006) deletion strain was constructed using the suicide plasmid pEX18-Ap [61]. To generate the gene deletion vector, the PCR-primer pair 5′-DsLOV + Ext (GAGCTCTCGGTCAGGTCCGGATATG-3′) and 3′-DsLOV + Ext (5′-AAGCTTGGAAGAGCACCGTGAACTC-3′) was used to amplify a 1853-bp PCR product which contains the *dslov* (dshi_2006) encoding sequence together with up- and downstream non-coding sequences to enable homologous recombination. The PCR product which contained recognition sites for restriction nucleases *Sac*I at the 5′-end and *Hind*III at the 3′-end was cloned into the corresponding sites of plasmid pEX18Ap. To interrupt the DsLOV encoding sequence, the gentamicin interposon cassette was amplified from plasmid pWKR202 [62] in a second PCR. At the same time, *Xag*I-recognition sequences (CCTNNNNNAGG) were introduced at both ends of the PCR product using primers 5′GmR *Xag*I: CCTCTTCGAGGTATCCATCAGGCAACGACG and 3′GmR *Xag*I: CCTTGCACAGGTACGGCCACAGTAACCAAC. The modified cassette was ligated into the *dslov Xag*I site of the recombinant pEX18-Ap plasmid. The successful insertion of the resistance cassette and its correct orientation (i.e. the same orientation as the DsLOV gene to avoid putative polar effects due to the interposon insertion) was further analyzed by restriction analysis and corroborated by DNA sequencing. The resulting pEX18Ap*∆dslov*::*Gm*^r^ plasmid was used for inactivation of the chromosomal *dslov* gene of *D. shibae* DFL12^T^. *E. coli* ST18 [63] cells were transformed with the suicide plasmid and used as donor strain for conjugative gene transfer to *D. shibae* DFL12^T^ wild-type cells [64]. Homologous recombination by a double-crossover event was confirmed by antibiotic resistance screenings and analytic PCR.

### Detection of photopigments

Photopigments were detected by their specific absorbance at 772 nm ±2 nm for bacteriochlorophyll *a* and at 482 nm ±2 nm for spheroidenone. *D. shibae* cultures were cultivated as described above for 44 hours. Afterwards, cells corresponding to an optical density of 1 at 660 nm were harvested by centrifugation (5000 × g, 10 min, 20°C). The photopigments were extracted from cell pellets by addition of 1 ml ethanol and subsequent incubation for 10 minutes at 50°C in the dark. Afterwards, the cell-debris was separated by centrifugation (9000 × g, 10 min, 20°C) and the supernatant was transferred into silica glass cuvettes. For spectral analysis, the absorbance was detected using the standard UV/Vis spectrometer Genesys6 (ThermoFischer Scientific, Madison, WI USA) with a resolution of 1 nm.

### Protein expression and purification

DsLOV was isolated from *E. coli* BL21(DE3) harboring pRhotHi-DsLOV *E. coli* cultivated in auto induction medium (TB, 0.2% lactose, 0.05% glucose, 50 μg/ml kanamycin) [4] and purified as described previously [24]. Briefly, the cell pellet (about 5 g cells, wet weight) was dissolved in 30 ml lysis buffer (50 mM NaH_2_PO_4_, 300 mM NaCl, 10 mM imidazole, pH 8.0). Cells were lysed by passing the cell suspension four times through a french pressure cell (Thermo Scientific, Waltham, MA). The soluble fraction and pellet were separated by centrifugation (9200 × g, 30 min). The His_6_-tagged DsLOV proteins were subsequently purified by metal affinity chromatography using a superflow Ni-NTA resin (QIAGEN, Hilden, Germany). Elution was performed with a gradient between washing buffer (50 mM NaH_2_PO_4_, 300 mM NaCl, 20 mM imidazole, pH 8.0) and elution buffer (50 mM NaH_2_PO_4_, 300 mM NaCl, 250 mM imidazole, pH 8.0). All purification steps were carried out at 4°C. The purity of the eluted fractions was anaylzed by SDS-PAGE. Pure fractions were pooled, and the elution buffer was exchanged to 10 mM Tris–HCl, pH 7.0 supplemented with 10 mM NaCl. Desalting was achieved using Vivaspins with a cut-off of 10 kDa. The maximal concentration of imidazole in all samples is estimated to be <10 μM. Hence, the presence of imidazol in the respective DsLOV preparations is expected to contribute only minimally to the observed adduct decay rates.

### Chromatographic techniques

Detection and quantification of the putative chromophores, i.e. FAD (flavin adenine dinucleotide), FMN (flavin mononucleotide), and riboflavin, respectively, were conducted as described previously [65].

### Spectroscopic techniques

All spectroscopic analyses were carried out under red light at 20°C, using either a Beckmann UV/Vis spectrophotometer DU-650 for absorbance and recovery kinetic measurements or by employing a Perkin-Elmer Luminescence Spectrometer LS 50B for fluorescence measurements as described previously [24]. For all measurements, the samples were diluted to an OD_450_ of 0.1 with 10 mM phosphate buffer, pH 8.0 supplemented with 300 mM NaCl. In order to generate the LOV proteins light state, the samples were illuminated for 30 s using a high-power blue-light (450 nm)-emitting LED (Luxeon Lumileds, Phillips, Aachen Germany). Dark state recovery was measured from illuminated samples by recording the absorption recovery at 485 nm. Reported adduct state lifetimes are derived from three independent measurements and were obtained by fitting experimental data to a mono-exponential decay function using software Origin (OriginLab corporation, Northhampton, MA, USA).

### SAXS measurements

SAXS data was measured at beamline BM29 at the ESRF using a X-ray wavelength of 1 Å at 10°C. Two samples of DsLOV with concentrations of 7.2 and 14.8 mg/ml were measured in phosphate buffer (10 mM NaH_2_PO_4_, 300 mM NaCl, pH 8.0). The buffer was measured before and after each protein sample. The samples were purged through a quartz capillary during X-ray exposure. For each sample ten frames with an exposure time of 2 sec each were recorded, and the frames without radiation damage were merged.

Data analysis was done using the ATSAS software package [66]. Measured data were scaled by the concentration, where the lower concentration was used for the smaller *q*-range, while the data at higher concentration was taken for the high *q*-range. The excluded volume was calculated with the program DATPOROD and the molecular mass was estimated by applying a division factor of 1.7 [66]. Theoretical scattering curves of the crystallographic structures of the monomer and the dimer were calculated and fitted to the experimental SAXS data using the program CRYSOL. The program EOM was used to generate 10000 native-like conformations of N-terminal residues 1–19 and the C-terminal His_6_-tag (LEHHHHHH), while the crystal structure of the dimer was held fixed. Consecutively, representative ensembles were selected, which best describe the measured data. The distance distribution function *P*(*r*) was determined using the program DATGNOM. *Ab initio* models were generated using the programs GASBOR and DAMMIF applying two-fold symmetry. In both cases 20 *ab initio* models were generated, averaged and the filtered model was used. The molecular mass was estimated from the excluded volume of the filtered DAMMIF *ab initio* model using a division factor of 2 [66]. The crystal structure of the dimer was aligned in the filtered model. The envelope function was determined using the SITUS package.

### Protein crystallization

The purified protein was concentrated to 25 mg/ml and crystallization set-ups were performed using the vapor-diffusion method. Crystals were grown in the dark using 1.8 μl sitting drops (0.9 μl purified protein + 0.9 μl reservoir solution) against 0.3 M MgCl_2_, 8% PGA-LM and 0.1 M sodium acetate, pH 5.0 at 19°C. Typically, crystals appeared after three weeks. No crystals could be grown in continuous blue-light conditions.

### Data collection and structure determination

Prior to cryo cooling, the crystals were soaked stepwise in reservoir solution containing up to 20% (v/v) glycerol. Both X-ray diffraction data were collected at 100 K, one under constant dark and one under constant light conditions. In the light condition, the crystals were exposed to blue-light prior to cryo cooling and in the subsequent steps. Please note that the crystals used for the datasets reported here were from the same drop and were stored in the dark at 100 K (in cryo tanks) until data collection. The X-ray diffraction data were recorded at beamline ID14-1 and ID23-1 of the European Synchrotron Radiation Facility (ESRF, Grenoble, France). The data collection strategies taking radiation damage into account were based on calculations using the program BEST [67]. Data processing was carried out using MOSFLM and SCALA, which is part of the CCP4 software package [68]. The dark state structure was determined by molecular replacement using PHASER [part of the PHENIX software suite [69]]. The search model was built by homology modeling *via* the ExPASy web server [70,71], based on the template of the PDB ID 2V0U (phototropin 1 from *Avena sativa*). Phase determination was followed by several cycles of automated model building and refinement using the PHENIX package. The model was further improved by manual rebuilding using the program COOT [72]. The refinement and rebuilding of the photoexcited structure was carried out with the finished dark state structure. For statistics on data collection and refinement refer to Table 1.

Unless otherwise indicated, figures were generated with MOLSCRIPT [73] and RASTER3D [74] using secondary structure assignments as given by the DSSP program [75].

#### PDB accession codes

Coordinates and structure factors for DsLOV (dark) and DsLOV (illuminated) have been deposited in the Protein Data Bank (http://www.pdb.org/pdb/) under accession codes 4KUK and 4KUO.

### Ethics statement

This research did not involve human subjects, human material, or human data.

## Additional file

Additional file 1: Table S1. Lifetimes for the dark recovery reaction in LOV proteins. **Figure S1.** Identification of three putative LOV genes in the genome of *D. shibae* DFL12^T^. **Figure S2.** Superposition of LOV domains. **Figure S3.** Differences between dark and photoexcited crystal structures of DsLOV. **Figure S4.** Single crystal microspectrometry of dark-grown DsLOV crystals.

## Abbreviations

ESRF: European Synchrotron Radiation Facility
FMN: Flavin mononucleotide
LOV: Light–oxygen–voltage
PAS: Per-ARNT-Sim
PCR: Polymerase chain reaction
PDB: Protein Data Bank
SAXS: Small angle X-ray scattering
