# Supplementary material for: Structure and function of a short LOV protein from the marine phototrophic bacterium Dinoroseobacter shibae
Source: BMC Microbiol. 2015 Feb 14;15:30. doi: 10.1186/s12866-015-0365-0 (PMC4335406; doi:10.1186/s12866-015-0365-0)
Supplement: Additional file 1: Table S1. — Lifetimes for the dark recovery reaction in LOV proteins. Figure S1. Identification of three putative LOV genes in the genome of D. shibae DFL12T. Figure S2. Superposition of LOV domains. Figure S3. Differences between dark and photoexcited crystal structures of DsLOV. Figure S4. Single crystal microspectrometry of dark-grown DsLOV crystals. [file 12866_2015_365_MOESM1_ESM.docx]

**Additional File 1:**

**A superfast recovering LOV protein from the marine phototrophic bacterium *Dinoroseobacter shibae***

Stephan Endres, Joachim Granzin, Franco Circolone, Andreas Stadler, Ulrich Krauss, Thomas Drepper, Vera Svensson, Esther Knieps-Grünhagen, Astrid Wirtz, Anneliese Cousin, Petra Tielen, Dieter Willbold, Karl-Erich Jaeger*, Renu Batra-Safferling*

**Table S1**: Lifetimes for the dark recovery reaction in LOV proteins

| LOV-Protein | τ_rec_ (sec) | Temperature | Reference |
| --- | --- | --- | --- |
| *Chlamydomonas reinhardtii* LOV1 | 204 | 25 °C | ([Losi et al., 2004](#_ENREF_4)) |
| *Chlamydomonas reinhardtii* LOV1 D31Q | 136 | 25 °C |  |
| *Chlamydomonas reinhardtii* LOV1 D31N | 131 | 25 °C |  |
| *Chlamydomonas reinhardtii* LOV1 R58K | 73 | 25 °C |  |
| *Chlamydomonas reinhardtii* LOV1 R58K/D31Q | 43 | 25 °C |  |
| *Bacillus subtilis* YtvA | 2700 | 25 °C | ([Losi et al., 2003](#_ENREF_5)) |
| *Neurospora crassa* VVD | 18 x 10^3^ | 4 °C | ([Schwerdtfeger and Linden, 2003](#_ENREF_8)) |
| *Pseudomonas putida* SB1 | 14.8 x 10^4^ | 20 °C | ([Jentzsch et al., 2009](#_ENREF_2)) |
| *Pseudomonas putida* SB2 | 137 | 20 °C |  |
| *Listeria monocytogenes* LOV-STAS | 91 | 20 °C | ([Chan et al., 2013](#_ENREF_1)) |
|  | τ_1/2_ (sec) |  |  |
| *Avena sativa* phot1 LOV1 | 11.5 | RT | ([Salomon et al., 2000](#_ENREF_7)) |
| *Avena sativa* phot1 LOV2 | 27 | RT |  |
| *Arabidopsis thaliana* phot1 LOV1 | 10 | RT | ([Kasahara et al., 2002](#_ENREF_3)) |
| *Arabidopsis thaliana* phot1 LOV2 | 29 | RT |  |
| *Arabidopsis thaliana* phot2 LOV1 | 13 | RT |  |
| *Arabidopsis thaliana* phot2 LOV2 | 5 | RT |  |
| *Oryza sativa* phot1 LOV1 | 27 | RT |  |
| *Oryza sativa* phot1 LOV2 | 43 | RT |  |
| *Oryza sativa* phot2 LOV1 | 18 | RT |  |
| *Oryza sativa* phot2 LOV2 | 14 | RT |  |
| *Chlamydomonas reinhardtii* Phot LOV1 | 168 | RT |  |
| *Chlamydomonas reinhardtii* Phot LOV2 | 20 | RT |  |

RT: room temperature

**Figure S1:**


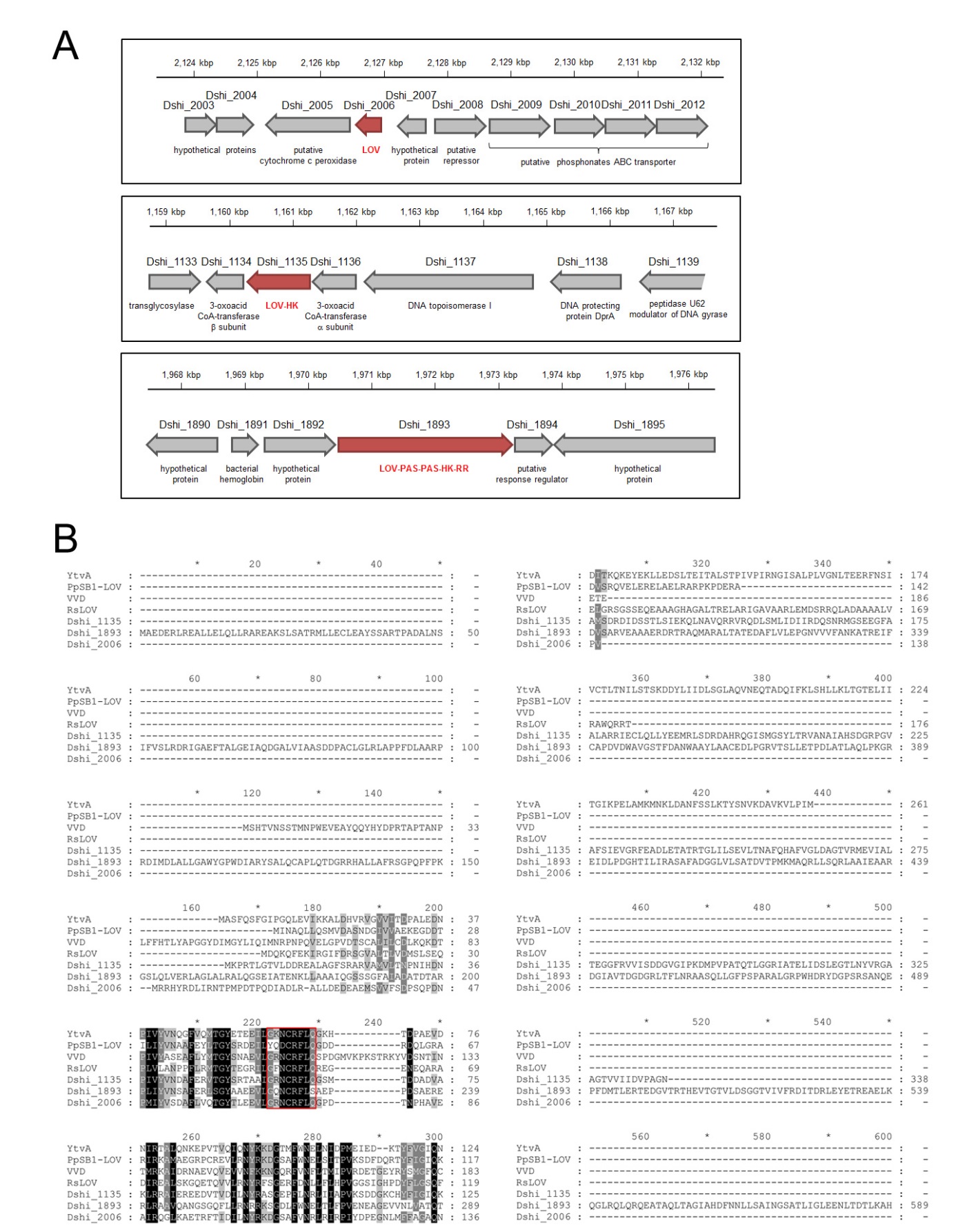


Identification of three putative LOV genes in the genome of *D. shibae* DFL12^T^. (**A**) Gene region of the respective LOV genes. The numbers indicate the base positions in the genome. The gene arrows show the orientation, length and assigned function of adjacent genes. LOV protein encoding genes are highlighted in red. (**B**) LOV sequence alignment. The amino acid sequences of the LOV proteins of *D. shibae* (Dshi_1135, Dshi_1893 and Dshi_2006) were aligned to the corresponding sequences of *B. subtilis* (YtvA), *P. putida* (PpSB1-LOV), *Neurospora crassa* (VVD) and *Rhodobacter sphaeroides* (RsLOV) using BioEdit (Version 7.1.3; http://www.mbio.ncsu.edu/BioEdit/bioedit.html). As matrix for the alignment, BLOSUM62 was used. Positions with identical amino acids are highlighted in black. Positions with 80 % and 60 % identity are marked in dark and light gray, respectively. The canonical LOV consensus sequence is marked in red. Sequence alignments of Dshi_2006 (designated as DsLOV in text), Dshi_1135 and Dshi_1893 with *B. subtilis* YtvA ([Losi et al., 2002](#_ENREF_32)), *P. putida* PpSB1-LOV ([Krauss et al., 2005](#_ENREF_26)), *N. crassa* VVD ([Heintzen et al., 2001](#_ENREF_18)) and *R. sphaeroides* RsLOV ([Metz et al., 2012](#_ENREF_35)) demonstrate that all of the three novel *D. shibae* LOV proteins contain the canonical LOV consensus sequence GXNCRFLQ which includes the photoactive cysteine (Salomon et al., 2000; Losi, 2004).

**Figure S2:**


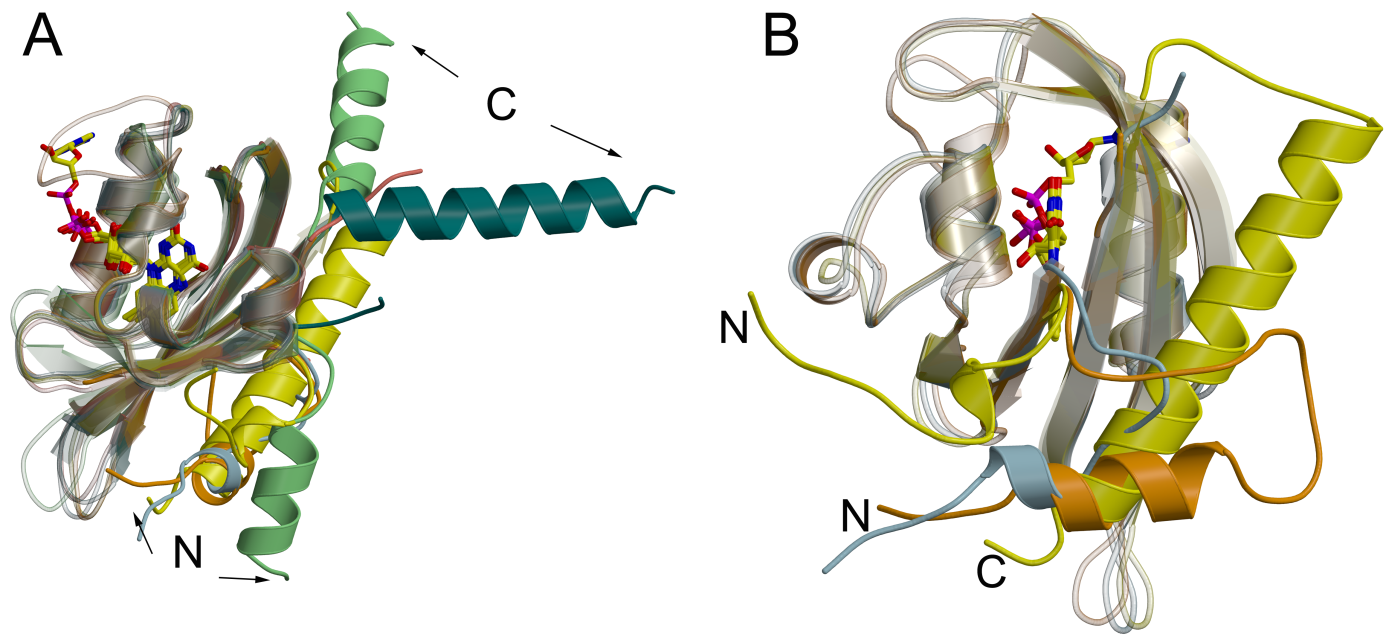


Superposition of LOV domains. (**A**) The selected LOV proteins DsLOV (light blue), *P*. *putida* SB1-LOV (PDB ID:1SW1; light green), *A*. *sativa* phot1-LOV2 (PDB ID: 2V0U; yellow), *N*. *crassa* Vivid (PDB ID: 2PD7; orange), *B*. *subtilis* YtvA (PDB ID: 2PR5; dark cyan), and *A*. *sativa* phot2-LOV1 (PDB ID: 2Z6D; salmon) were included in the analysis using the secondary structure matching program with residues from one protein chain in each case ([Winn et al., 2011](#_ENREF_46)). Differences are seen in the N- and C-terminus as well as in the loop regions indicated in the figure. (**B**) DsLOV (light blue), AsLOV2 (PDB ID: 2V0U; yellow), and *N*. *crassa* Vivid (PDB ID: 2PD7; orange), where N-cap residues of DsLOV and VVD follow the same trace.

The transparent regions correspond to the core domains. Chromophore molecules are shown as stick model colored by element: carbon, yellow; nitrogen, blue; oxygen, red; phosphate, purple.

**Figure S3:**


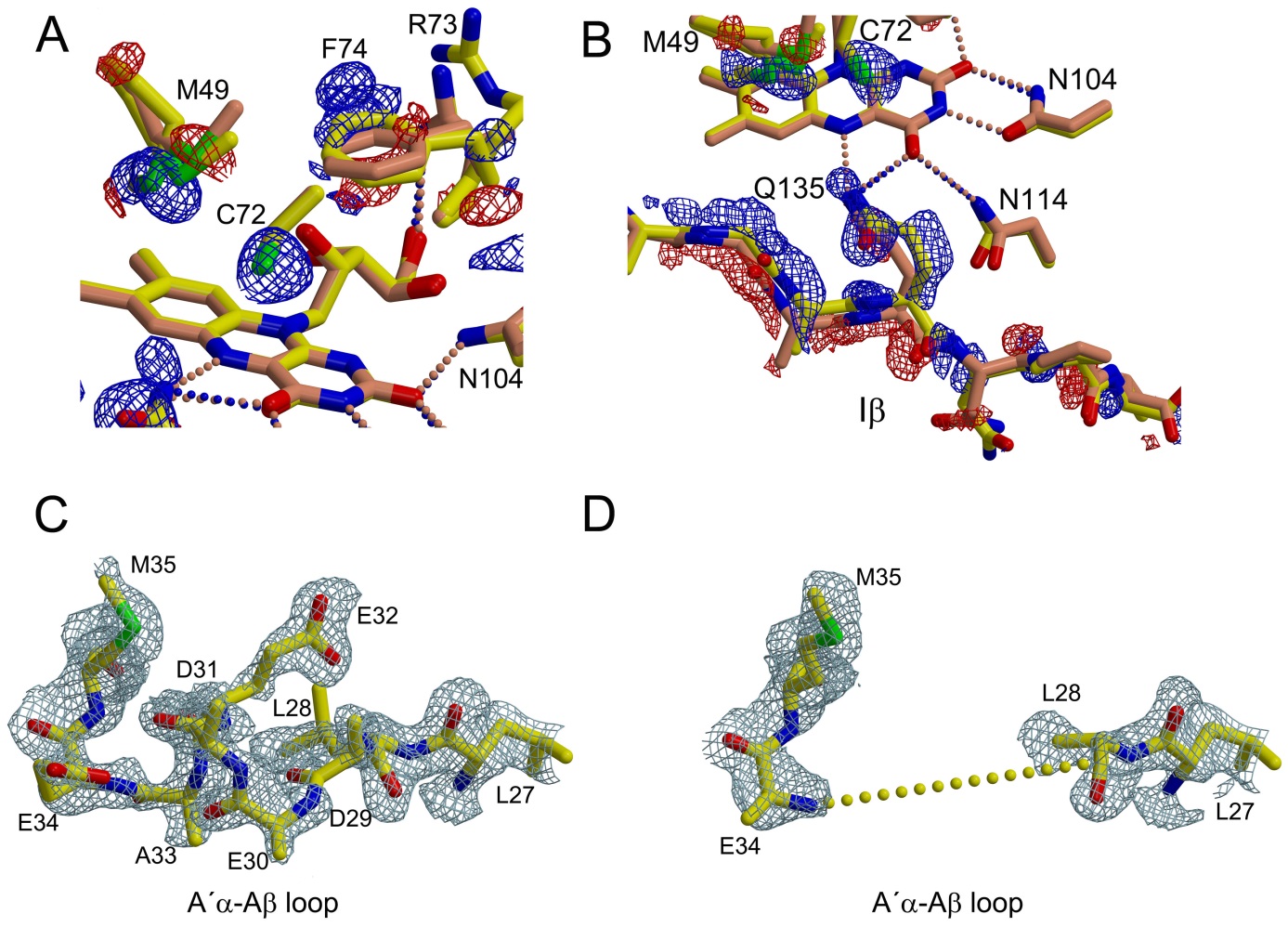


Differences between dark and photoexcited crystal structures of DsLOV. (**A** and **B**) Superposition of the two structures (color code: photoexcited -coral; dark -by element, carbon, yellow; nitrogen, blue; oxygen, red) where the difference electron density map F_dark_-F_light_ was phased by FOM weighted Φ_dark_. The density is contoured at 3 sigma level, where positive density is shown in blue and negative density is shown in red. The strength of sigma values at residues Cys72 (SG atom), Met49 (SD atom), Phe74 (ring) and Gln135 (OE1 atom) are +7.32, +8.17, +4.2 and +7.5, respectively. (**C** and **D**) Sigma-A weighted 2Fo-Fc electron density map of the residues in the N-terminal region including A´α helix (residue range 27 to 35) contoured at 0.7 σ. Panel C shows weak but continuous electron density in the given residue range of the dark adapted DsLOV. Panel D depicts the electron density of the photoexcited structure that shows disruption of the density even at such low σ level, indicating much higher flexibility in the N-terminal region.

**Figure S4:**


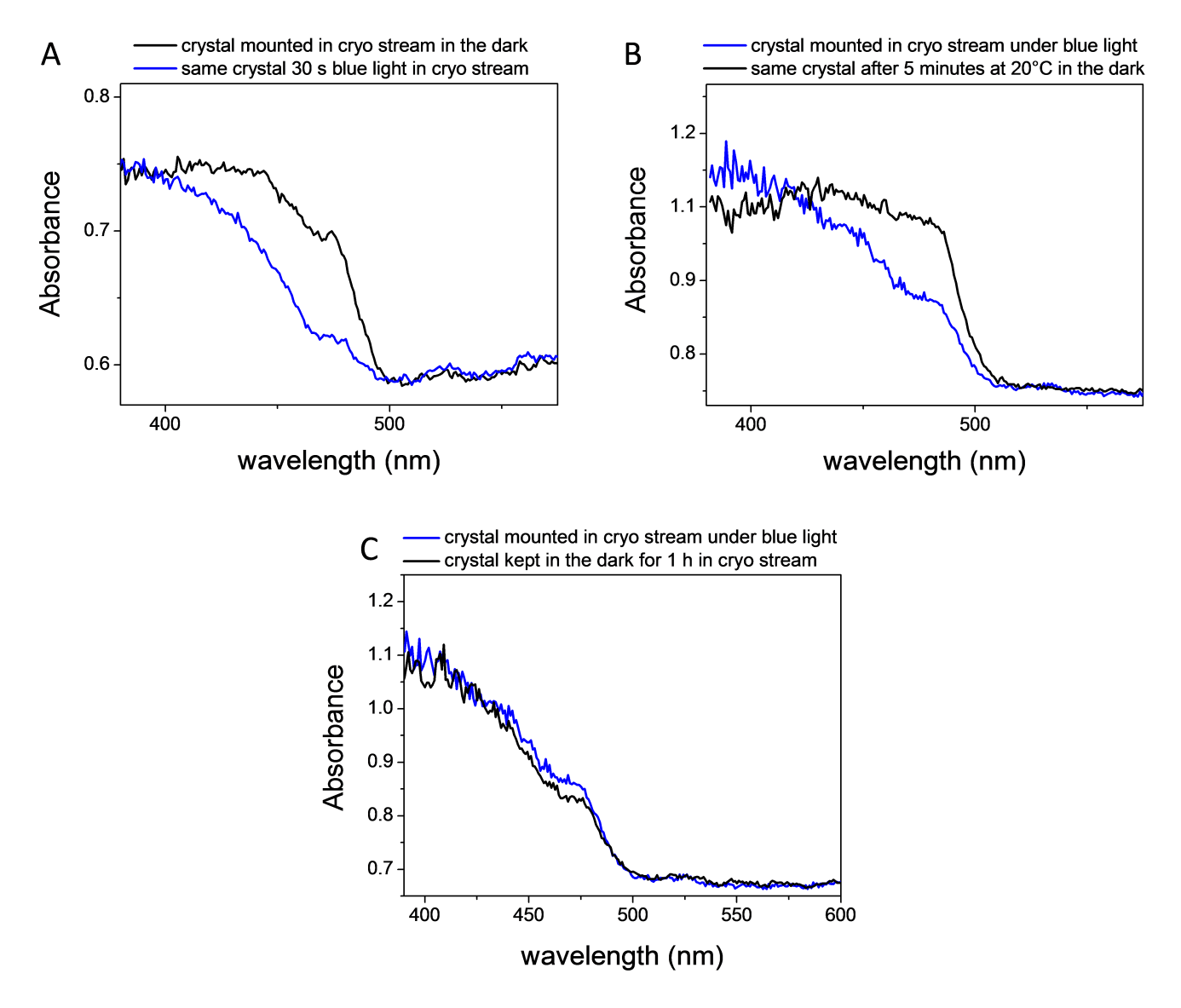


Single crystal microspectrometry. In order to verify photosensitivity of dark grown DsLOV crystals single crystal microspectrometry was used. (**A**) A dark grown DsLOV crystal was mounted at cryogenic temperature (100 K) in the dark (black line). Subsequently, a second spectrum on the same crystal was recorded after illumination for 30 s with blue light (blue line), demonstrating bleaching of the flavin specific absorption band in the blue region of the spectrum. (**B**) In order to verify reversibility of adduct formation, a dark grown DsLOV crystal was mounted under constant blue-light illumination at cryogenic temperatures (blue line). Subsequently, the cryo stream was blocked and the crystal was kept in the dark for 5 minutes at 20 °C (black line), demonstrating that adduct formation is fully reversible in dark grown DsLOV crystals at room temperature. (**C**) To verify trapping of the adduct state after illumination at cryogenic temperatures, a dark grown DsLOV crystal was mounted under constant illumination at cryogenic temperatures (blue line). Subsequently, the same crystal was kept in the cryo stream for 1 hour in the dark (black line), demonstrating trapping of the photoadduct at cryogenic temperatures.

**References to Additional File 1:**

Chan, R.H., Lewis, J.W., and Bogomolni, R.A. (2013). Photocycle of the LOV-STAS protein from the pathogen Listeria monocytogenes. Photochemistry and photobiology *89*, 361-369.

Heintzen, C., Loros, J.J., and Dunlap, J.C. (2001). The PAS protein VIVID defines a clock-associated feedback loop that represses light input, modulates gating, and regulates clock resetting. Cell *104*, 453-464.

Jentzsch, K., Wirtz, A., Circolone, F., Drepper, T., Losi, A., Gärtner, W., Jaeger, K.E., and Krauss, U. (2009). Mutual exchange of kinetic properties by extended mutagenesis in two short LOV domain proteins from Pseudomonas putida. Biochemistry *48*, 10321-10333.

Kasahara, M., Swartz, T.E., Olney, M.A., Onodera, A., Mochizuki, N., Fukuzawa, H., Asamizu, E., Tabata, S., Kanegae, H., Takano, M.*, et al.* (2002). Photochemical properties of the flavin mononucleotide-binding domains of the phototropins from Arabidopsis, rice, and Chlamydomonas reinhardtii. Plant physiology *129*, 762-773.

Krauss, U., Losi, A., Gärtner, W., Jaeger, K.-E., and Eggert, T. (2005). Initial characterization of a blue-light sensing, phototropin-related protein from Pseudomonas putida: a paradigm for an extended LOV construct. Phys Chem Chem Phys *7*, 2804-2811.

Losi, A., Polverini, E., Quest, B., and Gärtner, W. (2002). First evidence for phototropin-related blue-light receptors in prokaryotes. Biophys J 82, 2627-2634.

Losi, A., Quest, B., and Gärtner, W. (2003). Listening to the blue: the time-resolved thermodynamics of the bacterial blue-light receptor YtvA and its isolated LOV domain. Photochemical & photobiological sciences : Official journal of the European Photochemistry Association and the European Society for Photobiology *2*, 759-766.

Losi, A., Kottke, T., and Hegemann, P. (2004). Recording of blue light-induced energy and volume changes within the wild-type and mutated phot-LOV1 domain from Chlamydomonas reinhardtii. Biophysical journal *86*, 1051-1060.

Metz, S., Jager, A., and Klug, G. (2012). Role of a short light, oxygen, voltage (LOV) domain protein in blue light- and singlet oxygen-dependent gene regulation in Rhodobacter sphaeroides. Microbiology *158*, 368-379.

Salomon, M., Christie, J.M., Knieb, E., Lempert, U., and Briggs, W.R. (2000). Photochemical and mutational analysis of the FMN-binding domains of the plant blue light receptor, phototropin. Biochemistry *39*, 9401-9410.

Schwerdtfeger, C., and Linden, H. (2003). VIVID is a flavoprotein and serves as a fungal blue light photoreceptor for photoadaptation. The EMBO journal *22*, 4846-4855.

Winn, M.D., Ballard, C.C., Cowtan, K.D., Dodson, E.J., Emsley, P., Evans, P.R., Keegan, R.M., Krissinel, E.B., Leslie, A.G., McCoy, A.*, et al.* (2011). Overview of the CCP4 suite and current developments. Acta crystallographica Section D, Biological crystallography *67*, 235-242.
